# Retraction Note: KRAS activation in gastric cancer stem-like cells promotes tumor angiogenesis and metastasis

**DOI:** 10.1186/s12885-024-12164-2

**Published:** 2024-03-28

**Authors:** Changhwan Yoon, Jun Lu, Yukyung Jun, Yun-Suhk Suh, Bang-Jin Kim, Jacob E. Till, Jong Hyun Kim, Sara H. Keshavjee, Sandra Ryeom, Sam S. Yoon

**Affiliations:** 1 Department of Surgery, Columbia University Irving Medical Center, Milstein Hospital Building 7-002, 177 Fort Washington Avenue, 10032 New York, NY USA; 2 Department of Gastric Surgery, Fujian Medical University Union Hospital, Fuzhou, Fujian China; 3 Center for Supercomputing Applications, Korea Institute of Science and Technology Information, Division of National, SupercomputingDaejeon, Korea; 4 Department of Surgery, Seoul National University, Bundang Hospital, Seongnam, Korea; 5 Department of Surgery, Seoul National University College of Medicine, Seoul, Korea; 6 Department of Cancer Biology, Perelman School of Medicine, University of Pennsylvania, Philadelphia, PA USA; 7 Department of Biological Science, Hyupsung University, Hwasung-Si, Republic of Korea

**Retraction Note: BMC Cancer (2023) 23:690**

10.1186/s12885-023-11170-0

The Editors have retracted this article. After publication, concerns were raised regarding highly similar images in Fig. 5d (VEGF-A sh.KRAS-/sh.VEGF-A + and sh.KRAS+/sh.VEGF-A+). The authors have provided the original data to address these concerns; however, further checks by the publisher have found that a number of irregularities in the raw data files, as some of the images appear to be duplicated or overlap with samples representing different groups.

The Editors therefore no longer have confidence in the data presented in this article and its conclusions.

Yukung Jun and Sam S. Yoon agree to this retraction. None of the other authors have responded to any correspondence from the publisher about this retraction.

